# 伴有染色体碎裂化异常的多发性骨髓瘤3例

**DOI:** 10.3760/cma.j.issn.0253-2727.2022.12.010

**Published:** 2022-12

**Authors:** 春晓 吴, 招 曾, 琴荣 王, 丽君 文, 谦 王, 灵芝 颜, 松 金, 晓岚 施, 苏宁 陈, 琤琤 傅, 金兰 潘

**Affiliations:** 苏州大学附属第一医院，江苏省血液研究所，国家血液系统疾病临床医学研究中心，国家卫生健康委员会血栓与止血重点实验室，血液学协同创新中心，苏州 215006 Jiangsu Institute of Hematology, National Clinical Research Center for Hematologic Diseases, NHC Key Laboratory of Thrombosis and Hemostasis,Collaborative Innovation Center of Hematology, The First Affiliated Hospital of Soochow University, Suzhou 215006, China

多发性骨髓瘤（MM）是一种以单克隆浆细胞在骨髓中异常增殖为特征的血液肿瘤，可导致溶骨性破坏，常伴随高钙血症、肾功能不全、贫血和感染。在血清和（或）尿液中能检测出单克隆免疫球蛋白[1]。其发生率在所有肿瘤中约占1％，发生率随年龄增长逐渐升高[2]。尽管新药的应用已经使患者的生存率大大提高，但目前仍然是一种无法治愈的疾病[3]。原因之一为肿瘤浆细胞存在很大的异质性，主要由不同的细胞遗传学异常引起[4]。研究表明，MM的细胞遗传学异常包括原发性和继发性异常，原发性异常包括累及IGH的易位、超二倍体异常核型；继发性异常包括13q−、17p−、1q21扩增等不平衡异常。这些细胞遗传学异常在MM患者的预后评估中发挥重要作用。因为MM肿瘤浆细胞在体外培养不易分裂，常导致无分裂象或正常核型，近年来对MM细胞遗传学异常的检测已由原来的染色体核型分析技术逐渐过渡到荧光原位杂交（FISH）联合基因芯片（SNP-Array）技术，极大地提高了MM中细胞遗传学异常的检出率。基因芯片技术的应用弥补了FISH技术的局限性，发现了肿瘤浆细胞中FISH技术无法检测到的异常[5]–[14]。文献报道，基因芯片技术除检测到大量不平衡染色体重排以外，在2％～3％的原发性肿瘤中检测到染色体碎裂化异常发生[15]，该异常在MM中发生率约为1％，且认为该异常与疾病进展、复发及预后不良相关[5]。本研究从100例接受基因芯片技术检测的MM患者中筛选出3例伴有染色体碎裂化异常的病例，并对其临床资料进行了总结，现报道如下。

## 病例与方法

1. 病例：3例患者为2018年8月至2021年3月就诊于苏州大学附属第一医院，其中男2例，女1例，中位年龄56（52～73）岁。其中2例患者因腰背部疼痛就诊，1例患者因体检发现球蛋白异常升高就诊。生存期指自患者疾病确诊至死亡或末次随访日期，随访截止日期为2021年11月30日。

2. 染色体核型分析：应用骨髓细胞48 h培养法，按常规方法制备染色体，R显带技术进行核型分析，根据《人类细胞基因组学国际命名体制（ISCN）（2020）》进行核型描述，每例患者至少分析10个中期细胞。

3. 免疫分型：应用流式细胞术间接免疫荧光法和一组单抗检测肿瘤细胞的表面抗原。判断标准为：胞质抗原≥10％为阳性，细胞膜表面抗原≥20％为阳性。

4. FISH检测：应用磁珠分选技术（MACS）从骨髓标本中获得富集的CD138^+^浆细胞（PCs），CD138^+^浆细胞分选按照说明书（德国Miltenyi Biotec公司产品）进行。对CD138^+^浆细胞进行FISH检测，13q14、RB1、P53、1q21单色探针、IgH断裂重排探针、FGFR3/IgH、CCND1/IgH、MAF/IgH双色双融合探针均购自北京金菩嘉医疗科技有限公司，−20 °C保存。应用Olympus BX60荧光显微镜在DAPI/FITC/Texas Red三色滤光镜激发下观察间期和中期细胞荧光杂交信号。13q14缺失：两红色荧光信号者（2R）为缺失阴性细胞，一红色荧光信号者（1R）为缺失阳性细胞；RB1缺失：两绿色荧光信号者（2G）为缺失阴性细胞，一绿色荧光信号者（1G）为缺失阳性细胞；TP53缺失：两绿色荧光信号者（2G）为缺失阴性细胞，一绿色荧光信号者（1G）为缺失阳性细胞；1q21扩增：两红色荧光信号者（2R）为扩增阴性细胞，≥三红色荧光信号者（≥3R）为扩增阳性细胞；IgH断裂重排：两黄色荧光信号者（2Y）为断裂重排阴性细胞，一黄一红一绿色荧光信号者（1Y1R1G）为断裂重排阳性细胞；FGFR3/IgH、CCND1/IgH、MAF/IgH基因并置：两红两绿（2R2G）荧光信号者为基因并置阴性细胞，两黄一红一绿（2Y1R1G）或一黄一红一绿（1Y1R1G）荧光信号者为基因并置阳性细胞。每例至少分析300个间期细胞（不计重叠细胞），并计算阳性细胞的比例。应用Video Test FISH 2.0图像分析系统进行图像采集并保存。

5. 基因芯片（Cytoscan）：对CD138^+^浆细胞（PCs）进行基因芯片检测，行全基因组分析。如果异常累及>6条染色体，则被认为是染色体复杂化核型；如基因组涉及1～5条染色体，且每条染色体上存在≥10个断裂点，则被认为是染色体碎裂化异常[16]。

## 结果

1. 临床和血液学特点：例1因体检发现球蛋白异常升高（102 g/L）就诊，血清游离轻链示：游离κ 65mg/L，游离λ 4 975 mg/L，轻链比值0.013；血肌酐86.8 µmol/L，β_2_-微球蛋白（β_2_-MG）3.65 mg/L，骨髓穿刺提示异型浆细胞弥漫增生至30％～40％。例2因肋骨及腰背部疼痛就诊，血红蛋白73 g/L，血钙3.89 mmol/L；血清游离轻链示：游离κ 11.3 mg/L，游离λ 3 700 mg/L，轻链比值0.003；血肌酐 235 µmol/L，β_2_-MG>5.5 mg/L；骨髓穿刺提示浆细胞占62.5％，其中幼稚浆细胞占36％。例3因肋骨及腰背部疼痛就诊，血红蛋白84 g/L，血钙1.83 mmol/L；血清游离κ 250 mg/L，游离λ 8.61 mg/L，轻链比值29.04；尿液游离κ 2 875mg/L，游离λ 13.4 mg/L，轻链比值214.55。血肌酐77.68 µmol/L，β_2_-MG 4.71 mg/L；骨髓穿刺提示幼稚浆细胞占50％。3例患者全身CT平扫均显示多发溶骨性破坏，符合MM改变，均被诊断为MM。

2. 染色体核型分析：例1及例2进行了染色体分析，例1常规核型分析为正常核型，例2核型为伴有多种染色体数目增加或缺失及结构性异常的复杂混合核型：42-47, XX, add（X）（p23）, del（1）（q32q44）, del（3）（q21q27）, i（3）（p10）, −4, −7, +8, add（8）（p23）, add（8）（q24）, −9, add（11）（q23）, der（15）?t（1;15）（q11;p11）, −16, +19, −21, −21, +der（22）add（22）（p11）, +Mar1-4［CP10］。

3. 免疫分型：3例患者初诊时均进行了MM免疫分型检查，均有高比例单克隆性浆细胞，符合MM表型，例1免疫表型：CD138（+）、cλ（+）、CD56（±）、MUMI（+）；例2免疫表型：cλ（+）、CD56（+）、CD138（+）、CD38（+）、CD81（+）；例3免疫表型：cκ（±）、cλ（±）、CD56（+）、CD138（+）、CD27（+）、CD38（+）。

4. FISH：3例患者CD138（+）细胞均进行了MM相关FISH 检测，其中例1结果为1q21扩增阳性（具体阳性率不详），IGH重排阳性（具体阳性率不详）；例2 1q21扩增阳性（3R，70％），t（4;14）/FGFR3/IGH基因并置阳性（2Y1R1G，40％）；例3 13q14、Rb1、P53缺失阳性（1R 94％，1G 92％，1R 94％）。

5. 基因芯片：3例患者的骨髓细胞经CD138分选后，均进行了基因芯片检测，分子核型均显示为异常复杂核型，例1：45, XY, dup（1）（q21q32）, dup（1）（q32q44）, dup（2）（q31q37）, del（8）（p23p11）, dup（8）（q11q24）, dup（11）（p15q11）, del（11）（q13q14）, del（11）（q21q25）, -13；例2：48, XX, 1q+, 2p+, dup（3）（q25q26）, del（4）（q24q35）, dup（6）（p25p21）, dup（8）（q24）, dup（11）（q23q25）, dup（14）（q23q25）, +15, dup（16）（q21q23）, +19, del（21）（q11q22）；例3：54, XY, +3, +5, dup（6）（p25p22）, dup（6）（p22p11）, del（6）（q15q21）, del（6）（q21q27）, +7, del（8）（p23p12）, dup（8）（p11q24）, +9, dup（11）（q11q25）, del（12）（p13p11）, del（13）（q12q34）, +15, +15, del（17）（p13p11）, +19, +21，且3例患者均检测到染色体碎裂化现象。

例1的11号染色体有碎裂化现象，共涉及12个位点的基因拷贝数变异（CNV）：11q12.1q12.2、11q12.3q13.1、11q13.2q13.3、11q13.5q14.3、11q14.3、11q21q25的片段缺失，11p15.5q11、11q11q12.1、11q12.2q12.3、11q13.1q13.2、11q13.4q13.5和11q14.3q21的片段获得，片段大小为0.9～55.1 Mb。另外尚有1q21.1q32.1和1q32.1q44区域的片段获得，片段大小分别为57.4 Mb和45.7 Mb，前者累及CKS1B和SLAMF7基因。2q31.1q37.3片段缺失，大小为69.4 Mb。8p23.3p11.21缺失，8p11.2q11.1和8q11.21q24.3片段获得，大小分别为41.1 Mb、6.7 Mb、98 Mb。另有−13，共累及5条染色体，有19种CNV，其中10种CNV异常片段大小>5 Mb。

例2患者在病程中进行了2次基因芯片检测，均有4号染色体碎裂化现象。第一次结果中，4号染色体共涉及10个位点CNV异常：4p16.1、4q13.3、4q21.23、4q22.1、4q22.2、4q23q24、4q35.1q35.2的片段获得，4q21.3、4q22.3q23、4q24q35.1的片段缺失，片段大小为0.7～79.8 Mb。另外有1p12q44片段获得，大小为129 MB，累及CKS1B基因扩增。3号染色体共有8个区域有CNV异常，均为长臂3q：3q13.13q21.3、3q22.1q22.3、3q23、3q25.1q26.2、3q26.2q26.33、3q26.33q27.1片段获得，3q27.2q28、3q28q29片段缺失，片段大小为0.7～20 Mb。5q11.2、11q14.1、21q22.2q22.3、21q22.3片段缺失，大小为0.6～22.2 Mb。共累及15条染色体，有43种CNV，其中21种CNV异常片段大小>5 Mb。患者治疗后进行了第二次基因芯片检测，与第一次结果相比，无任何好转。4号染色体仍旧为碎裂化，共涉及11个位点CNV异常：4q13.3、4q21.23q21.3、4q22.1、4q22.2q22.3、4q23q24、4q35.1q35.2的片段获得，4q21.3q22.1、4q22.3q23、4q24q25、4q26q34.3、4q34.3q35.1的片段缺失，片段大小为0.7～64 Mb。1q21.1q44片段获得，大小为103.8 Mb，累及CKS1B基因扩增。共累及14条染色体，有34种CNV，其中19种的片段>5 Mb。

例3有6号染色体碎裂化现象，共涉及15个位点CNV异常，包括6p25.3p22.3、6p22.3p11.2、6q12、6q13q14.1、6q14.1、6q14.3q15、6q15、6q21等10个区域的片段获得，片段大小为0.5～35.3 Mb，6q14.1、6q14.1q14.3、6q15、6q15q21、6q21q27等5个区域的片段丢失，大小为2.2～61.8 Mb。8p23.3p12、8p12p11.23、8p11.23p11.21区域片段丢失，片段大小为3.7～29.1 Mb，8p12、8p11.21q24.3区域片段获得，片段大小为0.6～104.2 Mb，11p11.12q11和11q11q25区域片段获得，片段大小分别为4.8 Mb和79.5 Mb。12p13.33p11.1区域片段丢失，片段大小为33.7 Mb，累及CCND2和CD27基因丢失。13q12.3q34区域丢失，大小为83.7 Mb，累及Rb1基因丢失。17p13.3p11.2区域丢失，大小为16.8 Mb，累及P53基因丢失。另外有+3、+5、+7、+9、+15、+15、+19、+21染色体三体。共累及13条染色体，共33种CNV异常，其中18种>5 Mb。

6. 治疗经过及转归：例1确诊MM后，予4个疗程VRD方案（来那度胺25 mg/d，第1～14天；地塞米松20 mg/d，第1、2、4、5、8、9、11、12天；硼替佐米1.3 mg·m^−2^·d^−1^，第1、4、8、11天）化疗，浆细胞比例降至0.5％，后予自体造血干细胞采集，再予2个疗程VRD方案巩固治疗，自体造血干细胞移植后予RD方案（来那度胺10 mg/d，第1～14天；地塞米松10 mg，每周1次×4次）维持治疗，骨髓穿刺提示浆细胞比例回升至66％，全身低剂量CT显示多发溶骨性破坏，考虑疾病首次复发。后予DVD方案（达雷妥尤单抗16 mg/kg，每周1次×2次；硼替佐米1.3 mg/m^2^，第1、8天；地塞米松20 mg/d，第1、2、4、5、8、9天）化疗。2个疗程DVD方案后复查骨髓穿刺，异常浆细胞占53％，后予2次达雷妥尤单抗治疗后出现肌酐、球蛋白、β_2_-MG升高，高钙血症，考虑疾病进展。予硼替佐米（具体剂量不详），口服泊马度胺2 mg/d治疗，出现呼吸衰竭合并心力衰竭，目前为BCMA CAR-T细胞治疗后，一般情况差，WBC 3.65×10^9^/L，HGB 65 g/L，PLT 27×10^9^/L，随访时间39个月。

例2确诊后予4个疗程VRD方案（来那度胺10 mg，隔日给药1次，第1～14天；地塞米松20 mg/d，第1、2、4、5、8、9、11、12天；硼替佐米1.3 mg·m^−2^·d^−1^，第1、4、8、11天）化疗，同时血液透析治疗。患者在第1个疗程化疗期间出现肿瘤溶解综合征，急性左心衰竭，2个疗程后疗效评估血清学达非常好的部分缓解（VGPR），3个疗程后M蛋白较前升高，4个疗程结束后复查骨髓穿刺，原始幼稚浆细胞占68％，评估本病疾病进展（PD），后予IDD方案（伊沙佐米3 mg，每周1次×3次；脂质体阿霉素35 mg/d，第2天；地塞米松20 mg，每周1次×4次）挽救化疗无效，予达雷妥尤单抗（400 mg，每周1次，第1周；700 mg，每周1次，第2～4周）+地塞米松（40 mg，每周1次×4次）+环磷酰胺（50 mg/d×21 d）治疗，化疗期间再次出现肿瘤溶解，复查骨髓穿刺浆细胞比例降至1％，初步达VGPR。后予自体外周血造血干细胞移植，出院后疾病恶化死亡，总生存期为9个月。

例3确诊后予RD方案（来那度胺25 mg，隔日1次×3次；地塞米松5 mg/d×5 d）化疗，后予7个疗程VRD方案（来那度胺25 mg/d×21 d；地塞米松9.75 mg，每周1次×4次；硼替佐米1.3 mg/m^2^，每周1次×4次）治疗，目前一般情况尚可，随访时间为16个月。

## 讨论

高通量单细胞测序技术证明，MM发生和发展是一种分级模式，一般先由正常浆细胞获得一些重要的遗传学异常后，转化为意义未明的单克隆免疫球蛋白血症（MGUS），再发展为冒烟型骨髓瘤（SMM），最终发展为MM或浆细胞白血病。在疾病每个阶段，肿瘤浆细胞逐步积累更多的遗传学异常，导致疾病进展[4]。在MM各种预后分层体系中，细胞/分子遗传学异常占重要地位，如FISH检测到p53缺失、1q21扩增、1p32缺失、t（4;14）、t（14;16）、t（14;20）等为高危组异常，如有t（11;14）、t（6;14）及染色体三体等为标危组异常[6]–[10]。但上述异常并不能完全解释MM患者在病程进展和预后方面极大的异质性。文献报道，如果肿瘤浆细胞有复杂染色体重排，则进展较迅速，可缩短MGUS期，或直接由MGUS发展为MM[17]–[20]。目前认为有3种复杂染色体重排形式，包括染色体碎裂化，染色体异常合成、染色体复合体。染色体碎裂化为常见的复杂染色体重排之一[21]。

研究发现，染色体碎裂化事件是一种大规模但高度局部化的染色体断裂重排事件，是对同一时间内一次性获得的灾难性事件的响应[22]。Magrangeas等[23]认为MM中的碎裂化异常具有以下特点：与其他肿瘤相比，MM中碎裂化异常发生率较低；只有少数染色体发生拷贝数重排，包括1q、2、3、8q、10、16q等，其中只有1q和16q常被累及；被累及区域片段拷贝数常在1～2、2～4、3～4、1～6个之间波动；平均DNA断裂点<30个。本文3例发生碎裂化异常者分别在11、4、6号染色体。11号染色体有12个断裂点，1个位于11p，11个位于11q，发生丢失的片段拷贝数为0～1个，扩增的片段拷贝数为3～4个；例2患者两次均为4号染色体碎裂化异常，第1次10个断裂点，9个位于4q，1个位于4p；第二次11个断裂点，均位于4q。发生丢失的片段拷贝数为1～2个，扩增的片段拷贝数为3～4个。6号染色体有15个断裂点，13个位于6q，2个位于6p，发生丢失的片段拷贝数为1个，扩增的片段拷贝数为3～4个。3例碎裂化异常基本集中于染色体长臂，少数扩大至短臂，平均断裂点12个左右，异常区域片段拷贝数在1～2和3～4个之间波动，这些特点与文献相符，只是累及的染色体不在文献报道的常见染色体之中，可能与病例数较少有关。

染色体碎裂化异常导致染色体上大量基因的功能和表达异常，可能对特定的药物呈现耐药性。目前认为，在急性髓系白血病、急性淋巴细胞白血病、慢性淋巴细胞白血病中，染色体碎裂化与疾病的高度侵袭性和不良预后相关[24]–[26]。染色体碎裂化在MM中的意义尚未完全明确，有文献报道，MM患者同样有较差的无进展生存和总生存（OS）[19]–[20]。Kaur等[21]报道的初诊MM中6例（6/107）伴有碎裂化核型，发生率为5.6％，其中5例死亡，OS期仅8个月。Magrangeas等[23]发现，764例初诊MM中，10例为碎裂化核型，发生率1.3％，诊断后10个月，5例复发，4例死亡。Smetana等[10]报道，1例18号染色体碎裂化核型MM患者在诊断后23个月内快速复发并死亡。Lee等[22]研究发现，伴有染色体碎裂化异常核型的MM细胞株对硼替佐米耐药。

Lee等[22]研究发现，在耐药细胞株中，CNV发生率高，断裂点数目增加，而在药物敏感株中，断裂点数目少，表明染色体碎裂化与治疗反应相关。例2在初诊和治疗过程中各进行了1次基因芯片检测，第1次共有43种CNV，发生碎裂化的4号染色体有10种CNV异常，第2次共有34种CNV异常，但发生碎裂化的4号染色体有11种CNV异常，尽管第2次基因芯片结果累及的全基因组断裂点少于初诊时，但治疗过程中4号染色体的CNV却多于初诊时。另外，3例患者FISH检测均有高危遗传学异常，例1为1q21扩增，且IgH重排阳性；例2 1q21扩增合并t（4;14），根据mSMART3.0，为双打击MM，预后不良；例3 13q14，p53基因缺失。3例患者基因芯片同时检测到染色体碎裂化异常，可能表明染色体碎裂化异常易与其他高危遗传学异常并存，也表明染色体碎裂化异常为高危的遗传学预后因素之一。或许在以后的研究中，可以将该异常作为靶点进行靶向治疗。

国内外研究普遍认为MM中碎裂化异常发生率较低，本研究从100例做过基因芯片的MM患者中筛选出3例伴有碎裂化异常的病例，发生率3％。而Maclachlan等[27]报道，在752例初诊MM患者中，染色体碎裂化发生率为24％。我们推测可能有以下原因：①全基因组测序（WGS）或全外显子测序（WES）技术不仅从全基因组层面上检测出染色体片段的获得、复制、缺失等基因组拷贝数变化，且能检测出片段的倒位、平衡易位等信息；②为提高基因芯片检测结果的可信度，更好地为临床服务，我们将大于500 kb的片段变化列为异常CNV，而Maclachlan等[27]的文献中将大于50 kb的片段变化列为异常CNV；③本组研究病例数少于文献报道的病例数。尽管WGS/WES有技术上的优势，但目前国内外仍认为，比较基因组杂交（array-based comparative genomic hybridization, aCGH）或SNP-Array等技术是研究包括染色体碎裂化在内的拷贝数变异的经典且可靠的技术方法。

综上所述，尽管本组伴有染色体碎裂化的病例数较少，在初诊MM中发生率较低，但伴有该异常的MM患者有独特的特点：对各种治疗疗效不佳，疾病侵袭性更强，难以缓解，易进展，OS期较短，该异常似乎是另一种独立的高危遗传学预后因素。基因芯片技术可以检测到全面而详细的基因组拷贝数变化，如果将SNP-Array技术引入MM的常规诊断检测中，将对患者的疾病危险分层、预后判断产生巨大帮助。
